# Characterization of Odor-Active Compounds, Polyphenols, and Fatty Acids in Coffee Silverskin

**DOI:** 10.3390/molecules25132993

**Published:** 2020-06-30

**Authors:** Simone Angeloni, Serena Scortichini, Dennis Fiorini, Gianni Sagratini, Sauro Vittori, Silva D. Neiens, Martin Steinhaus, Valtcho D. Zheljazkov, Filippo Maggi, Giovanni Caprioli

**Affiliations:** 1 School of Pharmacy, University of Camerino, via Sant’ Agostino 1, I-62032 Camerino (MC), Italy; simone.angeloni@unicam.it (S.A.); gianni.sagratini@unicam.it (G.S.); sauro.vittori@unicam.it (S.V.); giovanni.caprioli@unicam.it (G.C.); 2 International Hub for Coffee Research and Innovation, 62020 Belforte del Chienti (MC), Italy; 3 School of Science and Technology, Chemistry Division, University of Camerino, V. S. Agostino 1, I-62032 Camerino (MC), Italy; serena.scortichini@unicam.it (S.S.); dennis.fiorini@unicam.it (D.F.); 4 Leibniz-Institute for Food Systems Biology at the Technical University of Munich, Lise-Meitner-Straße 34, 85354 Freising, Germany; silva.neiens@gmail.com (S.D.N.); martin.steinhaus@tum.de (M.S.); 5 Department of Crop and Soil Science, 431A Crop Science Building, 3050 SW Campus Way, Oregon State University, Corvallis, OR 97331, USA; Valtcho.jeliazkov@oregonstate.edu

**Keywords:** coffee silverskin, GC-O, aroma, volatile compounds, bioactive compounds, SPME

## Abstract

For the first time the volatile fraction of coffee silverskin has been studied focusing on odor-active compounds detected by gas chromatography-olfactometry/flame ionization detector (GC-O/FID) system. Two approaches, namely headspace (HS) analysis by solid-phase microextraction-gas chromatography-mass spectrometry (SPME-GC-MS) and odor-active compounds analysis by gas chromatography-olfactometry/flame ionization detector (GC-O/FID), have been employed to fully characterize the aroma profile of this by-product. This work also provided an entire characterization of the bioactive compounds present in coffee silverskin, including alkaloids, chlorogenic acids, phenolic acids, flavonoids, and secoiridoids, by using different extraction procedures and high performance liquid chromatography-tandem mass spectrometry (HPLC-MS/MS) system. Coffee silverskin was shown to be a good source of caffeine and chlorogenic acids but also of phenolic acids and flavonoids. In addition, the fatty acid composition of the coffee silverskin was established by GC-FID system. The results from this research could contribute to the development of innovative applications and reuses of coffee silverskin, an interesting resource with a high potential to be tapped by the food and nutraceutical sector, and possibly also in the cosmetics and perfumery.

## 1. Introduction

Coffee is one of the most consumed beverages in the world and an important agricultural product in the international trade. Coffee companies generate a significant amount of liquid and solid wastes (by-products); around 90% of the weight of coffee cherries (mostly pulp) is discarded during processing as agricultural waste or by-product [1]. Several authors have previously proposed different approaches to reuse the coffee by-products in order to reduce their disposal [2,3,4,5,6,7]. Among these by-products is coffee silverskin (CS), which is the major residue generated during the roasting process. It is a thin tegument that covers the coffee seeds, also known as coffee beans (CB). During roasting, CB expand and this thin layer is detached [8]. Although CS accounts for only a minimal fraction of the whole coffee cherry (1–2%), it contains high concentrations of dietary fiber (68−80%) and polysaccharides (60−70%). The total sugars content in CS varies greatly (2−12%) and it contains also fat (2−3%), protein (16−19%) and ash (5−7%) [1]. In addition, some bioactive compounds, e.g., caffeine, chlorogenic acids and melanoidins, responsible for different biological activities including antioxidant [5,9], occur in CS. Therefore, some authors proposed the use of CS as raw material for the recovery of functional compounds of potential interest. Indeed, CS is a rich source of soluble and insoluble dietary fibers (4 and 64%, respectively), which can be used for food enrichment [1]. Recent studies demonstrated that CS could be a valuable source of bioactive compounds such as melanoidins, caffeine and polyphenols, with potential applications of CS extracts as functional ingredients in cosmetic and nutraceutical formulations [8,10]. Other authors have suggested a potential use of this coffee residue as adsorbent material for removal of toxic metals from contaminated water [11], as a source of cellulose for paper production [5], and as a prospective ingredient in the food industry. Indeed, Martinez-Saez et al. [6] suggested the use of CS for the production of a novel beverage to be used in body weight control.

Several studies have reported the nutritional composition of CS and the content of bioactive compounds such as caffeine, chlorogenic acids, melanoidins and polyphenols [2,9,12]. Regarding polyphenols, the majority of the previously conducted research estimated their content in CS by measuring total phenolic and/or flavonoid content and none has investigated the individual concentrations of different polyphenolic and other bioactive compounds. Moreover, different authors have proposed innovative CS reuses and applications, but to the best of our knowledge, the aroma of this coffee by-product has not been reported. Therefore, there is a dearth of investigation focused on CS odorants which could be fascinating for food and food flavor industries. Furthermore, the characterization of odor-active compounds may foster role in the development of novel foods. Hence, the first objective of this research was to characterize the volatile fraction of CS employing two different techniques, i.e., analysis of odor-active compounds by gas chromatography-olfactometry/flame ionization detector (GC-O/FID) and analysis of headspace (HS) volatiles by solid-phase microextraction-gas chromatography-mass spectrometry (SPME-GC-MS). In the first case, after fractionation of the volatile extracts, the proposed chemical structures of odorants were confirmed by comparative analysis with reference compounds using comprehensive two-dimensional gas chromatography-mass spectrometry (GC×GC-MS) and the intensity of each odor was studied by Aroma Extract Dilution Analysis (AEDA). The second objective of this study was to provide an extensive characterization of the bioactive compounds present in CS. For this purpose, different extraction procedures, (i.e., liquid–solid extraction assisted and not by sonication, testing various solvents), were applied for the quantification of 30 bioactive compounds including alkaloids, chlorogenic acids, phenolic acids, flavonoids and secoiridoids, by using high performance liquid chromatography-tandem mass spectrometry (HPLC-MS/MS). Finally, the fatty acid composition was also investigated by GC-FID analysis. The present research will contribute to increase knowledge on volatile fraction, bioactive compound characterization and fatty acid composition of CS and, therefore, may facilitate the development of innovative applications of this product while reducing the amount of agricultural wastes that end up in landfills.

## 2. Results and Discussion

### 2.1. Odor-Active Compound Identification by GC-O/FID and GC×GC-TOF

**Figure 1:**
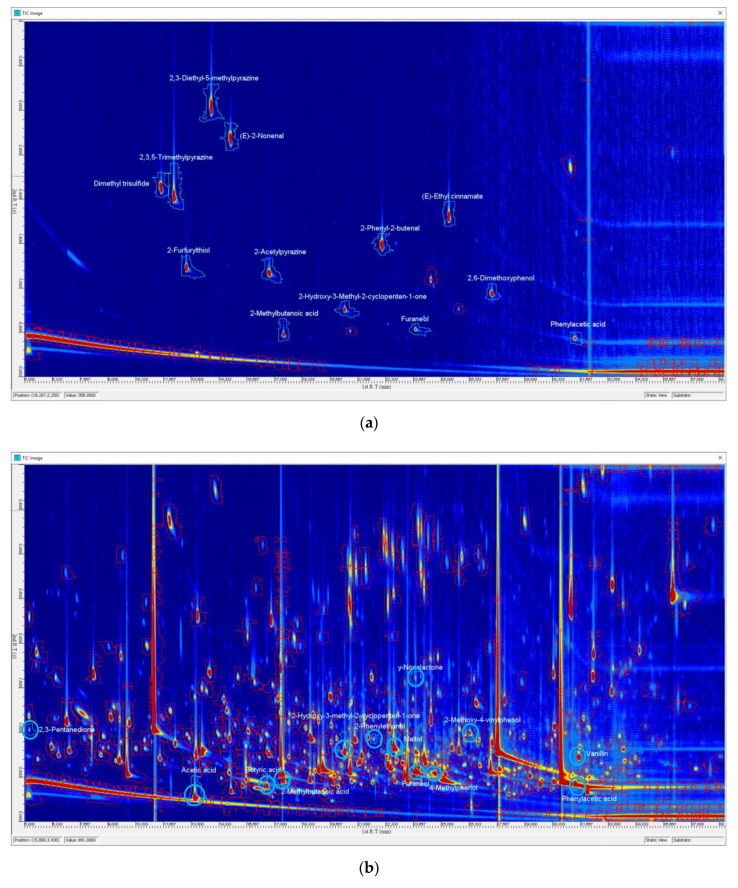
The two plots report the 2D-GC-MS total ion current chromatograms (TICs) of a mixture of thirteen reference compounds (**a**) and a sample of acidic volatiles (AV) fraction of coffee silverskin (**b**). The regions highlighted in sky blue represented the identified compounds.

The first identification step of odor-active compounds in CS and CB was performed by comparing the experimental linear retention indices (LRI) and the odor descriptions of the odorants present in different chromatogram regions, recorded during the AEDA, to outcomes obtained in previous works of coffee odorants [13,14], and to data compiled in the Leibniz-LSB@TUM Odorant Database [15]. In case of matching, authentic reference compounds were injected into GC-O/FID to confirm the proposed structures. The second step of identification was the comparison of the GC-O analysis of the concentrated volatile extracts and reference compounds using a second column with different polarity (DB-5). Finally, to confirm the proposed structures, samples were analyzed by GC×GC-TOF. Before injection, volatile extracts of CS and CB were separated into different fractions, as detailed below in Materials and Methods section. Each fraction was then analyzed by GC-O and by comprehensive two-dimensional GC-MS, together with reference compounds, to identify odor-active compounds. As an example, Figure 1 reports the TIC of 2D-GC-MS plots of a mixture of thirteen reference compounds (a) and a sample of acidic volatiles (AV) fraction of coffee silverskin (b).

### 2.2. Odor-Active Compounds in Coffee Silverskin (CS) by GC-O/FID and GC×GC-TOF Analysis

Table 1 shows all odors detected by GC-O and the assigned odorant structures with their LRI calculated on DB-FFAP and DB-5 columns, the odor descriptions and the flavor dilution (FD) factors for CS and CB. A case of unseparated odorants was observed for 3-methylbutanoic acid and 2-methybutanoic acid, which were characterized by a cheesy aroma. These two compounds were not separated on DB-FFAP column as well as on DB-5; the MS studies demonstrated the presence of both isomers in CS volatile fraction. Additionally, it was only possible to assert the presence of 2-methylbutanal or 3-methylbutanal.

A total of 40 odorants were identified in CS. The odorants with the highest FD factors were 4-hydroxy-2,5-dimethylfuran-3(2H)-one (furaneol) with 8192, 2-methoxy-4-vinylphenol (4-vinylguaiacol), 4096, and 2-methoxyphenol (guaiacol), 1024. The furanone possessed caramel-like notes, while the others were described as clove-like and phenolic. These volatiles were reported in several studies as important odor-active compounds, which contribute to coffee flavor [17,18,19,20,21]. High FD factors, from 512 to 128, were also found for 4-methyloctanoic acid, 512, *trans*-4,5-epoxy-(*E*)-2-decenal, 256, 4-hydroxy-3-methoxybenzaldehyde (vanillin), 256, 3-(methylthio)propionaldehyde (methional), 128, 2-isobutyl-3-methoxypyrazine, 128, 2-/3-methylbutanoic acid, 128, and phenylacetic acid, 128. 4-Methyloctanoic acid is a 4-alkyl-branched-chain fatty acid (vBCFA). Such compounds are responsible for the goaty-sheepy flavor of sheep and goat milk [22] and for the first time, we identified this fatty acid in coffee and coffee products. *trans*-4,5-Epoxy-(*E*)-2-decenal is an important volatile compound associated with metallic and blood-like odor; some behavioral studies reported that mammalian predators are as attracted by this single volatile compound as they are by the odor of real blood [23,24]. It has been identified in a coffee surrogate, namely chicory coffee [25] but, to the best of our knowledge, never in coffee or coffee beverages. 4-Hydroxy-3-methoxybenzaldehyde, 3-(methylthio)propionaldehyde, 2-isobutyl-3-methoxypyrazine, 2-/3-methylbutanoic acid and phenylacetic acid are common odorants reported in coffee beans and brews [17,19,26]. With FD factors from 64 to 16, fifteen volatiles were identified in CS: 2-furfurylthiol, 2,3-diethyl-5-methylpyrazine, (*E*)-2-nonenal, (*E*,*E*)-2,4-decadienal, 3-hydroxy-2-methyl-4-pyrone (maltol), *γ*-nonalactone, 2-isopropyl-3-methoxypyrazine, *γ*-decalactone, 3-methylindole (skatole), 2,3-butanedione, 1-octen-3-one, 2-acetyl-1-pyrroline, dimethyl trisulfide, acetic acid, 2-acetylthiazole and 4-methylphenol. 2-Furfurylthiol and 2-isopropyl-3-methoxypyrazine, are important volatiles in coffee, which possess (1) coffee-like, roasty and pungent odor, and (2) green, earthy odor [13,27]. Moreover, for 2-furfurylthiol a high Odor Activity Value (OAV) was reported in arabica and robusta coffee. [18,27]. The other odor-active compounds, with FD factors 64-16, have already been described in roasted beans and coffee beverages, except for *γ*-nonalactone. This lactone has never been detected in those matrices but some studies reported its presence in green beans [28,29]. Volatiles identified in CS with low FD factors (8-1) were 3-methyl-2-buten-1-thiol, 2,3,5-trimethylpyrazine, 3-ethyl-2,5-dimethylpyrazine, 2-acetylpyrazine, 2-acetyl-2-thiazoline, 2-hydroxy-3-methyl-2-cyclopenten-1-one, 2-phenyl-2-butenal, indole, 2-/3-methylbutanal, butanoic acid, 2-phenylethanol, 2,3-pentanedione, and 3,7-dimethylocta-1,6-dien-3-ol (linalool). All of these volatiles have been identified in previous works on coffee [30].

### 2.3. Odor-Active Compounds in Coffee Beans (CB) by GC-O/FID and GC×GC-TOF Analysis and Comparison with Coffee Silverskin (CS)

The GC-O/FID analysis of a concentrated volatile extract of coffee beans resulted in a large number of odors (about 150, 1 ≤ FD ≤ 16,384) and almost 2.5 times more than those of CS (63 odors, 1 ≤ FD ≤ 8192). For the direct comparison, it was necessary to keep the amount of the two matrices and the solvent/sample ratio consistent during the extraction process. Therefore, the stepwise diluted (1:2, 1:4, 1:8, 1:16) volatile extracts of CB were analyzed by GC-O/FID. A reasonable number of odors was found in the sixteen times diluted sample (1:16) hence the same was chosen as starting point of our studies. Our results showed that almost all odorants in CB occurred with higher FD factors than in CS (Table 1). Some volatiles were identified only in CB, such as 2-ethyl-5-methylpyrazine, 6-acetyl-2,3,4,5-tetrahydropyridine, 5-methyl-2-methoxyphenol and 3-ethylphenol. To the best of our knowledge, this is the first report on 5-methyl-2-methoxyphenol, a phenol derivate, and 6-acetyl-2,3,4,5-tetrahydropyridine, a pyridine derivate in CB. It has been reported that phenol compounds can be formed during roasting from quinic and caffeic acid and maybe also 5-methyl-2-methoxyphenol was formed from these molecules. [31,32]. 6-Acetyl-2,3,4,5-tetrahydropyridine is commonly present in the volatile fraction of baked products, e.g., bread and pretzels, and possesses a roasty and popcorn odor [33,34]. 3-Ethylphenol was described, after evaluation on the sniffing port, as phenolic, clove-like and has been already found in coffee [30,35]. On the other hand, three odorants, such as 1-octen-3-one, 2-phenylethanol and *γ*-decalactone were identified only in CS. The first odorant was described as mushroom-like, the second possessed sweet, honey-like notes and the third was described as peach-like and lemon-like. The most intensive odors in CB, in terms of FD factor were: 2-methoxyphenol, 16,384, 4-hydroxy-2,5-dimethylfuran-3(2H)-one, 16,384, and 2-methoxy-4-vinylphenol, 8192. These molecules were the most intense in CS as well. Other thirteen identified compounds occurred with high FD factors (from 1024 to 4096): 3-methyl-2-buten-1-thiol, 3-ethyl-2,5-dimethylpyrazine, butanoic acid, 2-/3-methylbutanoic acid, (*E*,*E*)-2,4-decadienal, *γ*-nonalactone, 2-isopropyl-3-methoxypyrazine, 2-furfurylthiol, 2,3-diethyl-5-methylpyrazine, 6-acetyl-2,3,4,5-tetrahydropyridine, 4-methyloctanoic acid, 3-(methylthio)propionaldehyde and 2-isobutyl-3-methoxypyrazine. Among these, 3-methyl-2-buten-1-thiol, 3-ethyl-2,5-dimethylpyrazine, 6-acetyl-2,3,4,5-tetrahydropyridine and butanoic acid may account for more intense roasty and popcorn aromas and also notes of thiol-like, and cheese-like in CB, since the ratio of CB and CS flavor dilution factors for the above-mentioned molecules were 128, 128, >2048, and 256, respectively. Other identified odorants with high FD factors (from 256 to 512) were 2,3-pentanedione, acetic acid, (*E*)-2-nonenal, 2-acetylthiazole, 2-acetyl-2-thiazoline, 5-methyl-2-methoxyphenol, 4-methylphenol, 3-methylindole, 2,3-butanedione, 2-hydroxy-3-methyl-2-cyclopenten-1-one, 3-hydroxy-2-methyl-4-pyrone, *trans*-4,5-epoxy-(*E*)-2-decenal, 3-ethylphenol, phenylacetic acid, and 4-hydroxy-3-methoxybenzaldehyde. The FD factor ratio of CB and CS demonstrated that butter-like 2,3-pentanedione, (ratio: 128), spicy and smoky 2-hydroxy-3-methyl-2-cyclopenten-1-one, (ratio: 64), phenolic and clove-like 5-methyl-2-methoxyphenol, (ratio >256), and phenolic and leather-like 3-ethylphenol (>512) were more intense in CB than in CS. In contrast, some odorants including two important actors of coffee flavor, were found with similar FD factors in both matrices: 4-hydroxy-2,5-dimethylfuran-3(2H)-one, 2-methoxy-4-vinylphenol, 4-hydroxy-3-methoxybenzaldehyde, *trans*-4,5-epoxy-(*E*)-2-decenal, 2-acetyl-1-pyrroline and 2-acetylpyrazine. In conclusion, our study revealed a potent odorant fraction in CS and therefore, this co-product can be considered as source of interesting and pleasant aroma and could be exploited in the food and other industries.

### 2.4. Volatile Substances Composition by HS-SPME-GC-MS Analysis

This study aimed to determine for the first time the volatile profile of CS. The main volatile substances detected by HS-SPME-GC-MS are presented in Table 2.

Several classes of compounds such as organic acids (especially short chain fatty acids), furans, furfurals, ketones, aldehydes, alcohols, pyridines, phenols, and lactones were detected in CS. The volatile substances qualitative and quantitative profile in roasted CB and their silverskin depends on the chemical composition of the raw seeds, their origin and maturation degree, and also on the roasting conditions [36]. About 1000 volatile organic compounds (VOCs) have been previously identified in different types of roasted CB with different analytical methods [13]. The classes of VOCs typically found are furans, pyrazines, ketones, phenols, alcohols, aldehydes, organic acids, esters, lactones, pyridines and sulfur compounds [21]. Their formation is usually due to the chemical processes involved during the roasting process and their presence and quantity is highly related to the roasting intensities. For example, some flavor compounds, such as furfural derivatives and furanones deriving from reactions involving sugars and lipids of green CB, seem to be in relatively high concentrations under mild roasting conditions (light roasting degree) than under higher roasting intensities (dark roasting degree). Pyridines and pyrroles, which can derive from the Maillard reaction, are mainly formed at high roasting intensities. Also, other VOCs formed from the degradation of chlorogenic acids (phenols and lactones) are found in greater amounts at high roasting temperatures [37].

The classes of VOCs detected in the present study were in accordance with the ones found in literature [36,37]. In fact, roasted coffee contains mainly furans, pyrazines, pyridines, alcohols, ketones, phenols, some aldehydes, and short chain fatty acids (SCFAs). In particular, acetic acid was the most abundant volatile compound detected by HS-SPME-GC-MS technique in terms of peak area percentage in the analyzed CS. Somporn et al. [36] found acetic acid as the most abundant VOC in roasted coffee. Then, other SCFAs were present in considerable amount in the sample under investigation, such as formic, propanoic, butanoic, 3-methylbutanoic, 3-methyl-2-butenoic acid, pentanoic,4-methylpentanoic acid, hexanoic, heptanoic, octanoic and nonanoic acids. In fact, during roasting process, carbohydrates like sucrose, begin to breakdown, leading to the formation of SCFAs such as acetic and formic. Depending on roasting conditions, acetic acid concentration can become 25 times higher than its initial green bean concentration. Overall acetic acid reaches its maximum level at light or medium roasts, then quickly dissipates as roasting progresses due to its high volatility [38]. At low concentrations SCFAs show pleasant and sweet-like sensory characteristics, but at higher amounts can impart ferment-like flavors. The presence of SCFAs, especially acetic, propionic and butanoic acids, makes CS a potential functional food. In fact, SCFAs have several beneficial effects: they are able to enhance the growth of beneficial intestinal bacteria, decrease blood pressure, and reduce fat absorption and the presence of pathogenic bacteria in the intestinal tract [39].

Regarding the other classes of VOCs detected in the sample, furans are generally associated with the aromas of nuts and caramel. For instance, the detected 2-furanmethanol is known to give bitter and toasted flavor, and was also found by Colzi et al. [40] as the most abundant compound in the lipid extract from the spent coffee in capsules. Then, furfural derivatives can be formed from monosaccharides and from the reaction between a sugar and an amino acid at high temperatures, suggesting that they are formed during the roasting step. The presence of furfural in the sample contributed to sweet, bread-like and caramel flavor [37]. Other classes of compounds important for CS aroma included aldehydes, such as hexanal, which is associated to grassy and green oily aroma and 2-methylbutanal and 3-methylbutanal, which are associated with malty aroma. Several pyrazines and pyridines, molecules responsible for toasted, nut and chocolate flavor notes [40], were identified by HS-SPME-GC-MS. Important for coffee aroma are also some phenolic compounds such as guaiacol (2-methoxyphenol), 4-vinylguaiacol, 4-ethylguaiacol and 4-hydroxy-3-methoxybenzaldehyde. These phenols arise from thermal degradation of chlorogenic acids, and these volatiles could have a role in flavor differentiation between arabica and robusta, as the two species contain significantly different amounts of chlorogenic acids [21]. In particular, guaiacol, 4-methylphenol, phenol, and 2-methylphenol were detected by HS-SPME-GC-MS system. Butyrolactone was also detected in the sample. Its presence in coffee volatiles has been reported in many studies since the 1960s. It possesses butter and coconut-like flavors and it may play an important role in the flavor of coffee and other food and beverages [37].

### 2.5. Fatty Acid Profile

The lipid content of CS investigated in this study was 7.49 ± 0.01 g/100 g. Table 3 presents the % fatty acid composition found in CS lipids.

Palmitic acid (C16:0) was the main fatty acid found (36%), followed by linoleic acid (C18:2 *n*-6, 28%), behenic acid (C22:0, 11%), and arachidic acid (C20:0, 9%). This profile is in agreement with the results obtained by Costa et al. [41]. In general, CS contained mainly saturated fatty acids (64%), followed by polyunsaturated (30%) and monounsaturated (6%) ones. A high concentration of SFAs together with a significant amount of phenolic compounds could limit the CS degradation due to lipid oxidation. The high content of linoleic acid (27.6 ± 0.10%), which is the second most abundant FA, could have positive effects on HDL cholesterol concentrations, reducing the risk of cardiovascular diseases [12].

### 2.6. Bioactive Compounds in Coffee Silverskin (CS)

Thirty bioactive compounds, including alkaloids, chlorogenic acids, phenolic acids, flavonoids, and secoiridoids were quantified in CS by using HPLC-MS/MS triple quadrupole. The analytical method was validated by investigating the linearity, reproducibility and the sensitivity as reported in a previous work [42]. Several extraction methods such as liquid–solid extraction assisted and not by sonication, and various solvents were evaluated for their ability to extract analytes from CS matrix. Table 4 reports the contents (in mg kg^−1^) of 30 analytes resulting from the eight different extraction methods. The highest content of total bioactive compounds was obtained with Method 4 (2005.613 ± 42.118 mg kg^−1^) and Method 2 (1910.549 ± 55.406 mg kg^−1^). In both cases ultrasound assisted extractions (UAE) were carried out but employing EtOH/H_2_O and H_2_O, respectively. Excluding the concentration of caffeine and chlorogenic acids to the total content of bioactive compounds, the highest concentrations were found for Method 4 (4.391 ± 0.228 mg kg^−1^) and 5 (4.226 ± 0.093 mg kg^−1^). 

Therefore, Method 4, i.e., an ethanol/water (70/30) extraction assisted by sonication, was the best procedure in term of extraction efficiency not only for caffeine and chlorogenic acids but also for polyphenols. This solvent was the best for the extraction of sixteen polyphenols from pulse samples as well [43]. Interesting levels of polyphenols were also obtained when the extraction was performed under acid condition (Method 5). This could be due to the prevention of polyphenols oxidation at low pH [43].

A total of 17 bioactive compounds were found in CS; caffeine (731.5−845.5 mg kg^−1^, RSD 2.5−5.5%) and chlorogenic acids (total contents: 974.6−1155.7 mg kg^−1^, RSD 1.8−6.3%) were the analytes present in the highest concentrations. Therefore, CS can be considered a good source to recover caffeine and chlorogenic acid and an interesting starting material for nutraceutical formulations. In fact, it has been reported that chlorogenic acids are an important class of biologically active dietary polyphenols, which are associated with several beneficial effects such as antioxidant activity, antibacterial, hepatoprotective, cardioprotective, anti-inflammatory, antipyretic, neuroprotective, anti-obesity, free radicals scavenger, and a central nervous system (CNS) stimulator [44]. The concentrations of caffeine and chlorogenic acid in this study were slightly lower than those reported previously in [44] and [40]. This could be due to various factors affecting the coffee sample such as coffee variety, processing method and roasting degree. In fact, the contents of chlorogenic acids and other polyphenols can be influenced by roasting degree and processing method [36,45] and the levels of caffeine can fluctuate depending on the used roasted beans from 0.1 to 2.0% (dry weight) [46]. The present work is one of the first on the quantification of unconjugated phenolic acids in CS; all 7 monitored phenolic acids were found in CS, as it was shown in Table 4. Vanillic (0.880−1.472 mg kg^−1^, RSD 2.1−3.6%) and caffeic acid (0.858−1.420 mg kg^−1^, RSD 3.2−6.2%) were the most abundant followed by syringic acid (0.094−0.356 mg kg^−1^, RSD 1.8−4.2%). Shikimic acid ranged from 0.198 to 0.520 mg kg^−1^ (RSD 3.1−4.6%); it is an important intermediate in the biosynthesis of lignin, aromatic amino acids and most alkaloids in plants and microorganisms [47]. This study is the first on the quantification of flavonoids including flavonols, flavan-3-ols, flavanone and anthocyanins, alkaloid (quinine), xanthone, iridoid and secoiridoids. Among these polyphenols, four molecules of flavonols such as rutin, hyperoside, kaempferol 3-glucoside, and quercitrin, were detected above their limit of detection (from 0.002 to 0.069 mg kg^−1^, RSD 3.2−5.8%). A flavanone, i.e., naringin (0.002−0.034 mg kg^−1^, RSD 4.6−6.3%), was found in the CS extracts of four extraction methods. Coffee silverskin can be considered not only a good source of caffeine and chlorogenic acids but also a resource of polyphenols such as phenolic acids and flavonoids.

## 3. Materials and Methods

### 3.1. Reagents and Standards

Cyanidin-3-glucoside chloride, delphinidin-3,5-diglucoside chloride, and kaempferol-3-glucoside were purchased from PhytoLab (Vestenbergsgreuth, Germany). The other 27 analytical standards of bioactive compounds were supplied by Sigma-Aldrich (Milan, Italy). Individual stock solutions of each analyte, at a concentration of 1000 mg L^−1^, were prepared by dissolving pure standard compounds in HPLC-grade methanol and storing them in glass-stoppered bottles at 4 °C. Afterwards, standard working solutions at various concentrations were prepared daily by appropriate dilution of the stock solution with methanol. HPLC-grade formic acid 99−100% was purchased from Merck (Darmstadt, Germany) and hydrochloric acid (HCl) 37% from Carlo Erba Reagents (Milan, Italy). HPLC-grade acetonitrile and methanol were supplied by Sigma-Aldrich (Milano, Italy). Deionized water was obtained from a Milli-Q Reagent Water System (Bedford, MA, USA). All other solvents and chemicals were analytical grade. All solvents and solutions for HPLC-MS/MS were filtered through a 0.2 µm polyamide filter from Sartorius Stedim (Göttingen, Germany). Before HPLC analysis, all samples were filtered with Phenex™ RC 4 mm 0.2 µm syringeless filter, Phenomenex (Castel Maggiore, Italy). Chloroform (CAS: 67-66-3) and sodium chloride (CAS: 7647-14-6) were purchased from Carlo Erba (Milan, Italy); while methanol (CAS: 67-56-1) was purchased from Fisher Scientific (Leicestershire, UK). Pure potassium chloride (CAS: 7447-40-7) was obtained from PanReac Quimica (Barcelona, Spain). Pure sodium chloride (CAS: 7647-14-5) was purchased by Carlo Erba (Milan, Italy). Anhydrous sodium sulphate (CAS: 7757-82-6) was purchased from Sigma-Aldrich (Milan, Italy). The potassium hydroxide (CAS:1310-58-3) was obtained from ProLabo (Fontenay-sous-Bois, France) and the Supelco 37 components, FAME Mix was purchased from Sigma-Aldrich (Milan, Italy). 

4-Hydroxy-3-methoxybenzaldehyde was purchased from Merck (Darmstadt, Germany), 2-methoxy-4-vinylphenol from Alfa Aesar (Karlsruhe, Germany), while other reference compounds of odor-active molecules were purchased from Sigma Aldrich (Taufkirchen, Germany). Dichloromethane, diethyl ether, and pentane were freshly distilled before use. Silica gel 60 (0.040−0.063 mm) was purchased from VWR (Darmstadt, Germany) and purified as described previously [48]. Mercurated agarose gel was prepared from Affi-Gel 10 (Bio-Rad, Munich, Germany) [49]. All other chemicals were analytical grade.

### 3.2. Coffee Silverskin and Coffee Beans Preparation and Odor-Active Compounds Extraction

Coffee silverskin (CS) and coffee bean (CB) samples, from 100% C*offea arabica* L. var. Catuai Rosso coming from Naranjo, Santa Cruz region (Guatemala), were provided by Perfero Caffè (Altidona, Italy) roasting company. The coffee berries were submitted to natural method which consisted of sun-drying the berries on raised bed with wire mesh (African bed) for 24 days. About 200 g of coffee silverskin were collected after the roasting process from 20 kg of green coffee. The roasting process was performed during 16 min and it reached the maximum temperature of 220 °C. Samples were kept in vacuum sealed bags at −20 °C.

Just before the extraction process, CS was immersed in liquid nitrogen and milled by GM 200 Retsch GrindoMix (time: 10 s; speed: 4000 rpm; in both rotation direction). CB was processed into a powder through 6875 Freezer/Mill High Capacity Cryogenic Grinder (SPEX SamplePrep, Stanmore, UK) using the following program: pre-cool, 2 min; run time, 1 min; cool time, 1 min; cycle, 3; rate, 14 cps. The volatile compounds in 20 g of CS or CB powder were extracted with 250 mL of dichloromethane under stirring at room temperature for 2 h. After filtration with filter paper, the volatile compounds were removed from the extract by Solvent Assisted Flavour Evaporation (SAFE) at 40 °C. The SAFE distillate was dried by adding anhydrous sodium sulfate and concentrated to 1 mL by using a Vigreux column (50 × 1 cm) and then a Bemelmans microdistillation device [50]. The concentrated volatile extracts were kept at −20 °C and the odor evaluation of a small amount of CS and CB extracts using fragrance test strips demonstrated the aroma equivalence to the starting materials.

### 3.3. Odorants Analysis: GC-O/FID and AEDA

A Trace GC Ultra gas chromatograph (Thermo Scientific, Dreieich, Germany) was equipped with a cold-on-column injector, a flame ionization detector (FID) and a tailor-made sniffing port [51]. Two types of fused silica columns were used for volatile separation: (a) DB-FFAP (30 m × 0.32 mm i.d., 0.25 µm film thickness); (b) DB-5 (30 m × 0.32 mm i.d., 0.25 µm film thickness) (both Agilent J&W, United States). The carrier gas was helium (He) at 60 KPa (DB-FFAP) and 65 KPa (DB-5) and the injection volume was 1 µL. The initial temperature of the oven was 40 °C (2 min), the gradients were at 6 °C/min to 230 °C for DB-FFAP and to 240 °C for DB-5, and held at 230 °C (DB-FFAP) and 240 °C (DB-5) for 5 min. The end of the analytical column was connected to a deactivated Y-shaped glass splitter which divided the column effluent in two equal parts that were directed via deactivated fused silica capillaries (50 cm × 0.25 mm i.d.) to the FID (250 °C) and the sniffing port (230 °C), respectively.

The concentrated volatile extracts of CS and CB were injected into the GC-O/FID system. The GC-O/FID analyses were carried out by three trained and experienced sniffers (two males, one female: aged 26−40) using the DB-FFAP column as well as the DB-5 column. The training consisted in weekly sensory evaluation sessions of reference odorants dissolved in water and the evaluation of reference mixtures by GC-O analysis. Each sniffer during the GC-O analysis, placed the nose in the region above the top of the sniffing port and evaluated the odor of the effluent. The positions and the descriptions of the odors were marked on the FID chromatogram registered by a recorder. On both columns, an experimental linear retention index (LRI) of each odor was calculated from their retention times and the retention times of adjacent n-alkanes by linear interpolation according to van Den Dool and Kratz [16]. Each sniffer repeated the analysis until data was reproducible.

Aroma Extract Dilution Analysis (AEDA) was performed by stepwise diluting, the concentrated coffee volatile extracts with dichloromethane (1:2, 1:4, 1:8, 1:16, 1:32, etc.). Each diluted sample was then injected into the GC-O/FID system using the DB-FFAP column. A flavor dilution (FD) factor was assigned to each odor-active compound, representing the dilution factor of the highest diluted sample in which the odorant was detected during GC-O/FID analysis by any of the three sniffers.

### 3.4. Fractionation of Coffee Silverskin and Coffee Beans Volatiles

The fractionation of volatile extracts was performed to simplify the CS and CB SAFE distillate and, consequently, to have less coelution during GC separation, aimed to facilitate the MS identification. Seven different fractions, i.e., acidic volatiles (AV), 5 neutral and basic volatiles (NBVA-E) and volatile thiols (VT), were prepared according to odor-active compounds commonly reported in coffee [19,21,52]. A SAFE distillate was prepared as described above and was extracted with aqueous sodium carbonate solution (0.5 mol L^−1^) in three portions (300 mL total). The organic phase (dichloromethane), containing the neutral and basic volatiles, was dried with anhydrous sodium sulfate and concentrated to 0.5 mL by using a Vigreux column and then a Bemelmans microdistillation device (NBV). The aqueous phase, containing the acidic volatiles, was washed with dichloromethane (50 mL) and then acidified with hydrochloric acid (32%) to pH 2. Acidic volatiles were re-extracted in three portions with dichloromethane (300 mL total) and the remaining water was removed by drying over anhydrous sodium sulfate. Finally, the organic phase was concentrated to 0.5 mL (AV). The fraction of NBV was further separated on a slurry of purified silica gel (9 g) in pentane using a water-cooled (12 °C) glass column (1 cm i.d.). The elution was carried out with five different mixtures of pentane and diethyl ether: A, 100:0; B, 90:10; C, 70:30; D, 50:50; E, 0:100 (v:v; 50 mL each). The eluate was collected in five portions of 50 mL and eluate portions were concentrated to 0.5 mL (NBVA-E). Another SAFE distillate was used to prepare a volatile thiol fraction by following a published procedure [48]. Briefly, the concentrated volatile extracts of CS and CB were applied onto mercurated agarose gel (1 g) in a glass column (0.5 cm i.d.). Then, the column was rinsed with dichloromethane (50 mL) and the volatile thiols were eluted with dithiothreitol (10 mmol/L) in dichloromethane (50 mL). The excess of dithiothreitol was removed by SAFE distillation, and the distillate was concentrated to 0.5 mL (VT).

### 3.5. GC×GC-TOF

The system consisted of a 6890 Plus gas chromatograph (Agilent Technologies, Waldbronn, Germany) and a Pegasus III TOFMS (Leco, Mönchengladbach, Germany). The GC was equipped with a KAS4 injector (Gerstel, Mühlheim/Ruhr, Germany). The injector was connected to a fused silica column, DB-FFAP, 30 m × 0.25 mm i.d., 0.25 μm film (Agilent). The end of this column was connected to a second fused silica column, DB-5, 2 m × 0.15 mm i.d., 0.30 μm film (Agilent). The front part of this column was passed through a liquid nitrogen-cooled dual-stage quad-jet thermal modulator (Leco), the major part was installed in a secondary oven mounted inside the primary GC oven, and the column end was connected via a heated (250 °C) transfer line to the MS inlet. Helium at 2 mL/min constant flow served as the carrier gas. The temperature of the first oven was 40 °C for 2 min, ramped up at 6°/min to 230 °C, and held for 5 min at 230 °C. The modulation time was 4 s. The temperature of the secondary oven was 70 °C for 2 min, ramped up at 6°/min to 250 °C, and held for 5 min at 250 °C. The mass spectrometer was operated in the EI mode at 70 eV with a scan range of *m*/*z* 35−350 and a scan rate of 100 spectra/s. Data evaluation was performed by means of GC Image (GC Image, Lincoln, NE, USA).

### 3.6. Volatile Substance Composition Analysis by HS-SPME-GC-MS

An aliquot of 0.5 g of triturated CS was weighed in a 10 mL screw cap vial with pierceable septum with 2 mL of water and 0.4 g of NaCl. Then the sample was conditioned at 40 °C for 20 min under agitation. A solid-phase microextraction fiber coated with 50/30 μm divinylbenzene/Carboxen/polydimethylsiloxane (DVB/CAR/PDMS), 1 cm long, was then exposed to the headspace of the sample for 1 h and then the fiber was retracted and exposed for 10 min into the hot injector (260 °C) of a 6850 gas chromatograph (Agilent, Santa Clara, CA, USA). The splitless injection (splitless time, 4 min) was used. The GC was coupled with a 5973 N mass spectrometer detector (Agilent). The GC was equipped with a capillary column coated with polyethylene glycol, DB-WAX, 60 m × 0.25 mm i.d., 0.25 μm film thickness (Agilent J&W). The end of the column was connected via a heated (260 °C) transfer line to the MS inlet. The carrier gas was helium at 1.2 mL/min. The initial oven temperature was 35 °C (min), the gradients were at 2.5 °C/min to 120 °C and 15 °C/min to 250 °C and held for 3.33 min. The mass spectrometer was operated in the EI mode at 70 eV with a scan range of *m*/*z* 29−400. Identification of eluted molecules was performed by comparison of the experimental linear retention indices, calculated with reference to linear alkanes, according to van Den Dool and Kratz [16], with those reported in literature, and with comparison of the experimental mass spectra with those of the NIST 08 library. Blank analysis was performed in order to identify contaminants.

### 3.7. Lipid Extraction from Coffee Silverskin

Silverskin lipids were obtained by Folch method extraction [53]. An aliquot of triturated sample (10 g) was dissolved in 160 mL of a solvent mixture of chloroform/methanol 2:1. The sample was homogenized for 3 min by Ultraturrax (Yellow Line DI 25s basic immersion-type homogenizer). The solution was filtered and the solvent was collected in a graduated 200 mL cylinder. The filter was washed with 40 mL of fresh solvent mixture, reaching a final volume of 200 mL. The solution was put in a separating funnel and washed with 40 mL of aqueous potassium chloride solution (0.88%). The organic phase was recovered in a flask and dried over anhydrous sodium sulfate. Subsequently, the solvent was removed with the use of a rotavapor until constant weight. Lastly, the lipid extract was recovered with 4 mL of chloroform and stored in a refrigerator at −20 °C.

### 3.8. Fatty Acid Composition Analysis by GC-FID

The fatty acids were derivatized to form the corresponding fatty acid methyl esters (FAMEs). A proper aliquot of the dried lipid extract (10 mg) of CS was dissolved in 1 mL of *n*-hexane and 100 μL of KOH 2N in methanol were added to the solution and shaken for 2 min with the help of a vortex device. Thereafter, the reaction was quenched by the addition of 1.5 mL of a saturated brine. The mixture was vortexed for 2 min and centrifuged for 5 min (5000 rpm). The upper layer was transferred to a 4 mL vial and anhydrous sodium sulfate was used to eliminate any remaining water. This mixture was further vortexed and centrifuged.

The supernatant was analyzed in a 6850 gas chromatograph (Agilent) equipped with a split/splitless injector (260 °C) and a flame ionization detector (250 °C). Compounds were separated using a fused-silica capillary column coated with 50% of cyanopropylphenyl-dimethylpolysiloxane, DB-225MS™, 30 m × 0.25 mm i.d., 0.25 μm film thickness (Agilent). The carrier gas used was hydrogen at a flow rate of 3.7 mL/min. The injection was performed in split mode (split ratio 30:1) and the injection volume was 1 μL. The initial oven temperature was 40 °C (3 min), ramped at 20 °C/min to 220 °C (5 min) and at 20 °C/min to 240 °C (1 min). FAMEs were identified by comparing retention times of analytes with reference solutions obtained from the FAMEs Mix. The results were expressed as relative percentage of each fatty acid, after correcting FAMEs peak areas using the theoretical response factors [54].

### 3.9. Coffee Silverskin Preparation and Bioactive Compounds Extraction

Just before the extraction process, CS was immersed in liquid nitrogen and milled by Ariete Blendy 570 grinder (Florence, Italy) through five cycles of 5 s. The extraction of the bioactive compounds was based on extraction methods optimized by Kamgang Nzekoue et al. [42] with some modifications. The following two sections describe the tested procedures: Ultrasound-assisted extraction (UAE) and liquid–solid extraction (LSE) without sonication. At the end eight extraction processes have been tested.

#### 3.9.1. Ultrasound Assisted Extractions (UAE)

10 g of CS powder were extracted with 100 mL of solvent using FALC ultrasonic bath (FALC, Treviglio, Italy) at a frequency of 40 KHz for 120 min at 20 °C. Seven solvents such as MeOH (Method 1), H_2_O (Method 2), MeOH/H_2_O (50/50, *v*/*v*) (Method 3), EtOH/H_2_O (70/30, *v*/*v*) (Method 4), MeOH pH 2 (Method 5), EtOH (Method 6), EtOH/MeOH (50/50, *v*/*v*) (Method 7), have been tested. Only for Method 5, after the extraction the pH of the sample was adjusted to 6 by adding 1 M KOH. After sonication, the sample was filtrated with filter paper and an aliquot of supernatant was collected, centrifuged at 13,000× *g* rpm for 10 min and filtrated with 0.2 µm syringeless filter. Eventually, it was injected into HPLC-MS/MS system.

#### 3.9.2. Liquid–Solid Extraction (LSE)

The extraction of 10 g of CS powder was performed with 100 mL of H_2_O (Method 8) keeping the sample in a water bath under magnetic stirring for 30 min at 80 °C. After extraction, the sample was cooled at room temperature and filtrated with filter paper. Then, an aliquot was centrifuged at 13,000× *g* rpm for 10 min, filtrated with 0.2 µm syringeless filter and injected into HPLC-MS/MS system.

### 3.10. HPLC-MS/MS Analyses

The HPLC-MS/MS studies were performed following a previous work of Kamgang Nzekoue et al. [42]. Briefly, the analysis was carried out by using a 1290 Infinity series liquid chromatograph (Agilent) and a 6420 Triple Quadrupole (Agilent) equipped with an electrospray ionization (ESI) source operating in negative and positive ionization mode. In fact, the instrument allowed to perform a one run with polarity switching without any problems. The separation of target compounds was achieved on a Kinetex PFP analytical column, 100 mm × 2.1 mm i.d., particle size 2.6 µm (Phenomenex, Torrance, CA, USA). The mobile phase was a mixture of water (A) and methanol (B) both with formic acid 0.1% at a flow rate of 0.2 mL min^−1^ in gradient elution mode. The composition of the mobile phase varied as follows: 0–2 min, isocratic condition, 20% B; 2–15 min, 80% B; 15–18 min, isocratic condition, 80% B; 18–23 min, 100% B; 23–35 min, 20% B. The injection volume was 2 µL. The temperature of the column was 30 °C and the temperature of the drying gas in the ionization source was 350 °C. The gas flow was 10 L/min, the nebulizer pressure was 172.369 kPa and the capillary voltage was 4000 V. Detection was performed in the Dynamic “multiple reaction monitoring” (Dynamic-MRM) mode and the Dynamic-MRM peak areas were integrated for quantification. The most abundant product ion was used for quantitation, and the other for qualification. The selected ion transitions and the mass spectrometer parameters including the specific time window for each compound (delta retention time) are reported in Table 5.

## 4. Conclusions

For the first time the volatile fraction of coffee silverskin has been studied using two approaches, i.e., HS analysis by SPME-GC-MS and odor-active compounds analysis by GC-O/FID system. Our studies demonstrated that coffee silverskin contains an interesting odor-active compound fraction with high similarity to coffee beans. Although beans are characterized by more complex and intense aroma, coffee silverskin remains an important co-product to be exploited in food industry, for instance in novel food production. In this context, it will assume an important role for further research on odorant quantification and sensory tests in order to determine the key aroma compounds. This work also provided an entire characterization of bioactive compounds together with the fatty acid composition. This research increased knowledge on coffee silverskin and it is hoped that the results can contribute to the development an its original application in food and nutraceutical sector. In addition, in the optical of more sustainable economy, this work could encourage the use of coffee silverskin in certain industrial fields and therefore, it can contribute to the decrease in coffee waste and the disposal costs. This may lead to an eco-friendlier coffee production and consumption.

## Figures and Tables

**Table 1:** Odors and odorants described and identified in coffee silverskin (CS) and coffee beans (CB) with their linear retention indices (LRI) and flavor dilution (FD) factors measured in DB-FFAP.

No.	Odorant	Odor	LRI ^b^		FD ^c^	
			FFAP	DB-5	CS	CB
1	2-/3-Methylbutanal ^a^	malty	953	659	4	64
2	2,3-Butanedione ^a^	butter-like	980	606	16	512
3	2,3-Pentanedione	butter-like	1055	700	2	256
4	3-Methyl-2-buten-1-thiol ^a^	garlic-like, thiol	1097	819	8	1024
5	1-Octen-3-one ^a^	mushroom-like	1293	983	16	<1
6	unknown	roasty, fatty	1298	-	<1	256
7	2-Acetyl-1-pyrroline ^a^	roasty	1330	922	16	32
8	Dimethyl trisulfide	spicy, cabbage, sulfurous	1364	967	16	128
9	2-Ethyl-5-Methylpyrazine	roasty, nutty	1380	989	<1	128
10	2,3,5-Trimethylpyrazine	fatty, roasty, earthy	1398	1007	8	128
11	unknown	roasty, fatty	1402	-	<1	256
12	2-Isopropyl-3-methoxypyrazine ^a^	green, earthy	1420	1093	32	2048
13	2-Furfurylthiol ^a^	pungent, coffee-like	1427	907	64	2048
14	3-Ethyl-2,5-dimethylpyrazine	roasty, popcorn, earthy	1439	1084	8	1024
15	Acetic acid	pungent, vinegar	1443	624	16	256
16	3-(Methylthio)propionaldehyde ^a^	cooked potato	1452	904	128	4096
17	2,3-Diethyl-5-methylpyrazine	roasty, fatty	1480	1157	64	2048
18	2-Isobutyl-3-methoxypyrazine ^a^	green pea-like, green bell pepper	1512	1177	128	4096
19	(*E*)-2-Nonenal	greasy, green, roasty	1524	1157	64	256
20	3,7-Dimethylocta-1,6-dien-3-ol	sweet, fruity, citrus	1536	1102	1	64
21	6-Acetyl-2,3,4,5-tetrahydropyridine ^a^	roasty, popcorn	1556	1054	<1	2048
22	unknown	roasty, green	1572	-	<1	2048
23	unknown	fatty, roasty	1581	-	32	<1
24	Butanoic acid	sweaty, cheese	1618	819	4	1024
25	2-Acetylpyrazine	roasty	1625	1020	8	16
26	2-Acetylthiazole	roasty	1638	1020	16	256
27	2-/3-Methylbutanoic acid	cheese-like	1658	860	128	1024
28	unknown	meaty, greasy	1693	1212	32	32
29	unknown	silverskin-like	1718	-	512	512
30	unknown	spicy	1724	-	32	256
31	2-Acetyl-2-thiazoline	fatty, roasty	1750	1100	8	256
32	unknown	caramel-like	1787	-	<1	128
33	(*E*,*E*)-2,4-Decadienal	meaty, gravy-like	1800	1315	64	1024
34	2-Hydroxy-3-methyl-2-cyclopenten-1-one	spicy, burnt paper, smoky	1827	1029	8	512
35	unknown	pungent, spicy, clove-like	1832	-	128	256
36	2-Methoxyphenol	clove-like, phenolic	1854	1087	1024	16,384
37	2-Phenylethanol	sweet, honey	1906	1111	4	<1
38	2-Phenyl-2-butenal	green, phenolic	1926	1278	8	64
38	5-Methyl-2-Methoxyphenol ^a^	phenolic, clove-like	1937	1185	<1	256
40	unknown	smoky	1957	1123	64	<1
41	3-Hydroxy-2-methyl-4-pyrone	caramel-like	1969	1115	64	512
42	*trans*-4,5-Epoxy-(*E*)-2-decenal ^a^	metallic	1997	1377	256	512
43	*γ*-Nonalactone	fruity, coconut	2026	1364	64	1024
44	4-Hydroxy-2,5-dimethylfuran-3(2H)-one	caramel-like	2032	1064	8192	16,384
45	unknown	burnt paper, seasoning	2059	-	128	256
46	4-Methylphenol	fecal, horse stable-like, pee-like	2079	1080	16	256
47	4-Methyloctanoic acid ^a^	goaty, sheepy	2091	-	512	2048
48	*γ*-Decalactone ^a^	peach-like, lemon-like	2147	1471	32	<1
49	unknown	rubber-like	2178	-	32	<1
50	3-Ethylphenol	phenolic, leather-like	2181	1169	<1	512
51	2-Methoxy-4-vinylphenol	clove-like	2197	1315	4096	8192
52	unknown	seasoning, phenolic	2269	1357	128	<1
53	Indole	fecal	2448	1298	8	64
54	3-Methylindole	fecal	2493	1387	32	256
55	Phenylacetic acid	honey	2555	1259	128	512
56	4-Hydroxy-3-methoxybenzaldehyde	vanilla, chocolate-like	2572	1402	256	512
57	unknown	saliva-like	2619	-	64	128

^a^ GC×GC-MS analysis did not result in a clear mass spectrum, but comparison of linear retention index and odor quality with respective data of an authentic reference compound allowed for unequivocal structure assignment. ^b^ Experimental linear retention index (LRI), calculated according to van Den Dool and Kratz [16]. ^c^ Only more powerful odors (unknown chemical structure) with, for CS, FD > 16 and for CB, FD > 128 are reported.

**Table 2:** Volatile compounds detected in coffee silverskin (CS) by HS-SPME-GC-MS; their experimental linear retention indices (LRI) on a polyethyleneglycol coated column, their abundances in terms of peak areas percentages, and their relative standard deviations (RSD %, *n* = 3).

Compound Detected ^a^	LRI ^b^ (exptl)	LRI ^c^ (lit)	Area %	RSD %
2-Methylbutanal	903	910	1.44	14.77
3-Methylbutanal	907	913	2.86	12.76
1-Chloropentane	927	941	0.74	10.22
2-Ethylfuran	938	951	0.22	13.26
Pentanal	966	971	1.56	5.18
Hexanal	1069	1077	8.47	2.35
1-(1-Cyclohexen-1-yl)-ethanone	1109	/	0.25	6.61
3-Methyl-1-pentene	1116	/	0.22	22.82
Heptanal	1173	1179	1.68	0.45
D-Limonene ^d^	1180	1190	0.87	12.27
2-Methyl-2-butenal	1189	1129	0.25	10.43
2-Hexenal	1208	1209	0.65	18.39
2-Pentylfuran	1218	1230	1.80	10.33
Styrene ^d^	1240	1247	0.79	16.55
1-Pentanol	1252	1255	0.73	11.35
2,6-Dimethylpyridine	1255	1248	0.59	19.12
Methylpyrazine	1259	1262	0.41	8.88
1-Octen-3-one	1292	1298	0.46	13.78
(*E*)-2-Heptenal	1314	1318	1.73	2.66
4,6-Dimethylpyrimidine	1318	1363	0.12	11.91
3-Methyl-2-buten-1-ol	1321	1317	0.25	7.30
2-Heptanol	1323	1318	0.42	7.86
2,6-Dimethylpyrazine	1324	1330	0.55	0.37
Ethylpyrazine	1327	1338	0.26	11.96
6-Methyl-5-hepten-2-one	1329	1333	1.01	9.79
2,3-Dimethylpyrazine	1342	1345	0.31	14.72
1-Hexanol	1355	1352	0.33	7.73
2-Ethyl-5-methylpyrazine	1381	1382	0.72	1.48
2-Ethyl-6-methylpyrazine	1388	1389	0.87	12.52
2-Ethyl-3-methylpyrazine	1401	1406	0.32	9.68
Trimethylpyrazine	1403	1404	0.22	5.32
(*Z*)-3-Ethyl-2-methyl-1,3-hexadiene	1405	/	0.22	3.04
(*E*)-2-Octenal	1422	1425	1.78	2.99
Acetic acid	1441	1431	3.68	12.88
*trans*-Linalool oxide ^d^	1443	1460	7.15	4.29
Furfural	1450	1452	5.56	3.57
4-Ethenyl-1,4-dimethylcyclohexene	1456	/	0.46	15.96
*cis*-Linaloloxide ^d^	1471	1475	2.17	6.24
Formic acid	1492	1492	1.11	16.27
Benzaldehyde	1509	1520	4.86	2.33
Furfuryl acetate	1530	1539	0.24	2.79
Propanoic acid	1536	1535	1.15	1.03
3,7-Dimethylocta-1,6-dien-3-ol	1555	1554	0.41	1.34
5-Methyl-2-furfural	1570	1579	4.45	0.95
3-Furoic acid	1572	/	0.34	5.78
3-Methoxypyridine	1584	1581	0.33	11.85
6-Methyl-3,5-heptadiene-2-one	1591	1590	1.08	4.74
3-Methyl-2-cyclohexen-1-one	1593	1592	0.76	5.61
1,5-Dimethyl-2-pyridone	1600	/	0.65	3.61
2-Acetyl-5-methylfuran	1607	1608	0.59	10.28
Pyrrole-2-carboxaldehyde	1611	1610	1.88	5.62
Butyrolactone	1620	1618	0.54	7.11
Butanoic acid	1625	1628	0.38	6.54
Acetophenone	1645	1640	1.89	2.59
2-Furanmethanol	1659	1662	1.86	2.46
3-Methylbutanoic acid	1668	1665	3.55	13.46
1-Adamantol ^d^	1682	1661	0.61	2.15
2,6,6-Trimethyl-2-cyclohexene-1,4-dione	1690	1680	0.51	7.05
2-Methoxypyrimidine	1709	/	0.35	14.04
5-Methyl-2-furanmethanol	1719	1720	0.24	18.74
3,4-Dimethyl-2,5-furandione	1725	/	0.52	18.53
Pentanoic acid	1734	1733	1.32	3.97
3,5,5-Trimethylcyclohexene	1744	/	0.30	15.36
Methyl salycilate ^d^	1770	1771	0.52	8.45
1-(4-Methylphenyl)-ethanone	1773	1771	1.16	8.63
3-Methyl-2-butenoic acid	1789	1802	0.71	0.57
4-Methyl-pentanoic acid	1797	1795	0.22	13.01
Phenylethyl acetate	1813	1807	0.26	10.02
1-Furfurylpyrrole	1822	1817	0.26	7.75
Hexanoic acid	1841	1846	4.06	8.01
2-Methoxyphenol	1858	1853	0.73	9.51
Benzyl alcohol	1877	1879	1.13	8.87
Phenylethyl Alcohol	1916	1913	3.64	5.84
Heptanoic acid	1946	1942	0.72	4.78
1-(1H-pyrrol-2-yl)-ethanone	1974	1972	1.09	5.76
Phenol	1996	1999	0.48	8.31
1H-Pyrrole-2-carboxaldehyde	2028	2032	1.26	11.98
Octanoic acid	2051	2060	0.75	15.38
2-Methylphenol	2077	2060	0.26	3.61
4-Methylphenol	2085	2079	0.25	10.72
Nonanoic acid	2158	2168	0.45	9.43

^a^ Compounds reported are those which had peak area values higher than 500,00. ^b^ Experimental linear retention index calculated according to van Den Dool and Kratz [16]. ^c^ Linear retention indices reported in literature (NIST 2017). ^d^ D-Limonene, (R)-4-isopropenyl-1-methyl-1-cyclohexene; Styrene, ethenylbenzene; *trans*-Linalool oxide, *trans*-2-methyl-2-vinyl-5-(1-hydroxy-1-methylethyl)tetrahydrofuran; *cis*-Linaloloxide, 5-(3,3-dimethyloxiran-2-yl)-3-methylpent-1-en-3-ol; 1-Adamantol, tricyclo[3.3.1.1(3,7)]decan-1-ol; Methyl salycilate, methyl 2-hydroxybenzoate.

**Table 3:** Average fatty acid % composition ± standard deviation (*n* = 3) of coffee silverskin (CS) lipids.

Fatty Acid Composition (%)	
C14:0	1.28 ± 0.03
C15:0	0.21 ± 0.01
C16:0	35.64 ± 0.19
C17:0	0.14 ± 0.01
C18:0	6.38 ± 0.09
C18:1 *n*-9	5.77 ± 0.10
C18:1 *c*-11	0.64 ± 0.01
C18:2 *n*-6	27.62 ± 0.10
C18:3 *n*-6	0.75 ± 0.01
C18:3 *n*-3	0.88 ± 0.04
C20:0	8.62 ± 0.04
CLA ^a^	0.33 ± 0.01
C22:0	10.70 ± 0.06
C24:0	1.03 ± 0.03
SFA	64.01 ± 0.12
MUFA	6.41 ± 0.08
PUFA	29.58 ± 0.04

^a^ Conjugated Linoleic Acid (C18:2, *c-*9, *t-*11).

**Table 4:** Contents (mg kg^−1^) of bioactive compounds in CS extracted with eight processes.

		Extraction Solvents ^a^							
No	Analytes ^b^	MeOH	H_2_O	MeOH/ H_2_O	EtOH/ H_2_O	MeOH pH 2	EtOH	MeOH/ EtOH	H_2_O ^c^
1	Shikimic acid	0.328	0.520	0.390	0.502	0.323	0.198	0.273	0.252
2	Gallic acid	0.209	0.310	0.223	0.250	0.182	0.190	0.174	0.161
3	Loganic acid	n.d. ^d^	n.d.	n.d.	n.d.	n.d.	n.d.	n.d.	n.d.
4	5-CQA	940.420	940.400	961.800	985.705	854.909	903.109	910.350	900.785
5	Swertiamarin	n.d.	n.d.	n.d.	n.d.	n.d.	n.d.	n.d.	n.d.
6	Gentiopicroside	n.d.	n.d.	n.d.	n.d.	n.d.	n.d.	n.d.	n.d.
7	(*+*)-Catechin	n.d.	n.d.	n.d.	n.d.	n.d.	n.d.	n.d.	n.d.
8	Del-3,5-diglu	n.d.	n.d.	n.d.	n.d.	n.d.	n.d.	n.d.	n.d.
9	Sweroside	n.d.	n.d.	n.d.	n.d.	n.d.	n.d.	n.d.	n.d.
10	3-CQA	112.530	100.800	111.960	128.512	100.636	105.027	102.275	101.869
11	Caffeine	761.800	845.280	785.280	845.516	768.436	800.727	731.500	817.385
12	Cya-3-glu	n.d.	n.d.	n.d.	n.d.	n.d.	n.d.	n.d.	n.d.
13	Vanillic acid	1.410	0.880	1.090	1.472	1.320	1.364	1.342	1.138
14	Caffeic acid	1.250	0.858	1.030	1.361	1.420	1.127	1.125	1.038
15	(*−*)-Epicatechin	n.d.	n.d.	n.d.	n.d.	n.d.	n.d.	n.d.	n.d.
16	Syringic acid	0.203	0.094	0.113	0.295	0.356	0.184	0.169	0.156
17	*p*-Coumaric acid	0.128	0.100	0.120	0.150	0.221	0.116	0.107	0.098
18	Ferulic acid	0.183	0.152	0.183	0.204	0.139	0.166	0.152	0.141
19	3,5-diCQA	27.480	21.007	25.208	41.489	19.097	22.982	22.900	20.138
20	Quinine	n.d.	n.d.	n.d.	n.d.	n.d.	n.d.	n.d.	n.d.
21	Naringin	n.d.	0.002	0.003	0.009	0.034	n.d.	n.d.	n.d.
22	Rutin	0.021	0.042	0.051	0.024	0.039	0.019	0.017	0.016
23	Hyperoside	0.004	0.010	0.012	0.003	0.009	0.004	0.003	0.003
24	*Trans*-cin acid	0.066	0.055	0.066	0.066	0.082	0.060	0.055	0.050
25	Resveratrol	n.d.	n.d.	n.d.	n.d.	n.d.	n.d.	n.d.	n.d.
26	Amarogentin	n.d.	n.d.	n.d.	n.d.	n.d.	n.d.	n.d.	n.d.
27	Kae-3-glu	0.044	0.037	0.045	0.052	0.069	0.040	0.037	0.034
28	Quercitrin	0.002	0.002	0.002	0.003	0.032	0.002	0.002	0.002
29	Quercetin	n.d.	n.d.	n.d.	n.d.	n.d.	n.d.	n.d.	n.d.
30	Isogentisin	n.d.	n.d.	n.d.	n.d.	n.d.	n.d.	n.d.	n.d.
Tot bio compounds		1846.079	1910.549	1887.576	2005.613	1747.304	1835.315	1770.481	1843.266

^a^ Each sample was analyzed in triplicate (*n* = 3) and RSD values were from 2.3 to 9.8%. ^b^ Del-3,5-diglu, Delphinidin-3,3-diglucosiede; Cya-3-glu, Cyanidin-3-glucoside; *Trans*-cin acid, *Trans*-cinnamic acid; Kae-3-glu, Kaempferol-3-glucoside; Tot bio compounds, Total bioactive compounds. ^c^ Liquid–solid extraction without sonication. ^d^ n.d., not detectable.

**Table 5:** High performance liquid chromatography-tandem mass spectrometry (HPLC-MS/MS) acquisition parameters, working as Dynamic “Multiple Reaction Monitoring” mode, including retention time (Rt) and delta retention time (Δ Rt) for each transition.

No.	Compounds	Precursor ion (*m*/*z*)	Product Ion (*m*/*z*)	Fragmentor (V)	Collision Energy (V)	Polarity	Retention Time (Rt) (min)	Delta Retention Time (Δ Rt)
1	Shikimic acid	173	173	87	0	Negative	1.40	3
			-	-	-	-		
2	Gallic acid	169	125 ^a^	92	12	Negative	2.37	3
			51		36			
3	Loganic acid	375	213 ^a^	126	8	Negative	3.13	3
			113		16			
4	5-Caffeoylquinic acid	353	191 ^a^	102	12	Negative	3.58	3
			179		12			
5	Swertiamarin	419	179 ^a^	100	4	Negative	4.89	3
			89		16			
6	Gentiopicroside	357	177 ^a^	50	10	Positive	5.33	3
			73		28			
7	(*+*)-Catechin	289	245 ^a^	121	8	Negative	5.48	3
			109		24			
8	Delphinidin-3,5-diglucoside	463	300 ^a^	165	24	Negative	5.64	3
			271		48			
9	Sweroside	403	125 ^a^	102	12	Negative	5.95	3
			179		4			
10	3-Caffeoylquinic acid	353	191 ^a^	92	12	Negative	6.22	3
			85		48			
11	Caffeine	195	138 ^a^	107	20	Positive	6.50	3
			110		24			
12	Cyanidin-3-glucoside	449	287 ^a^	121	20	Positive	6.50	3
			403		16			
13	Vanillic acid	167	108 ^a^	78	16	Negative	6.70	3
			152		8			
14	Caffeic acid	179	135 ^a^	87	12	Negative	6.87	3
			134		24			
15	(*−*)-Epicatechin	289	245 ^a^	126	8	Negative	7.03	3
			109		20			
16	Syringic acid	197	182 ^a^	92	8	Negative	7.48	3
			123		20			
17	*p*-Coumaric acid	163	119 ^a^	83	12	Negative	8.47	3
			93		32			
18	Ferulic acid	193	134 ^a^	88	12	Negative	9.16	3
			178		8			
19	3,5-Dicaffeoylquinic acid	515	353 ^a^	117	8	Negative	9.82	3
			191		28			
20	Quinine	325	79 ^a^	135	44	Positive	10.1	5
			81		32			
21	Naringin	579	271 ^a^	210	32	Negative	10.17	3
			151		48			
22	Rutin	609	300 ^a^	195	40	Negative	10.34	3
			271		50			
23	Hyperoside	463	300 ^a^	160	24	Negative	10.43	3
			271		44			
24	*Trans*-cinnamic acid	149	131 ^a^	44	8	Positive	10.79	3
			77		36			
25	Resveratrol	227	185 ^a^	131	12	Negative	10.92	3
			143		20			
26	Amarogentin	585	227 ^a^	145	16	Negative	11.05	3
			245		16			
27	Kaempferol-3-glucoside	447	284 ^a^	163	24	Negative	11.24	3
			227		50			
28	Quercitrin	447	300 ^a^	155	24	Negative	11.24	3
			301		16			
29	Quercetin	301	151 ^a^	126	16	Negative	13.03	3
			179		12			
30	Isogentisin	257	242 ^a^	116	16	Negative	16.31	3
			214		24			

^a^ These product ions were used for quantification, the others to confirm the analytes.
